# Angoff anchor statements: setting a flawed gold standard?

**DOI:** 10.15694/mep.2017.000167

**Published:** 2017-09-21

**Authors:** Steven Ashley Burr, Daniel Zahra, John Cookson, Vehid Max Salih, Elizabeth Gabe-Thomas, Iain Martin Robinson

**Affiliations:** 1 Peninsula Schools of Medicine & Dentistry; 2 Hull York Medical School

**Keywords:** Angoff, standard setting, assessment

## Abstract

This article was migrated. The article was marked as recommended.

The Angoff standard setting method depends fundamentally on the conceptualisation of an anchor statement. The precise wording and consequent interpretation of anchor statements varies in practice. Emphasis is often placed on standard setting judges’ perceptions of difficulty for a candidate subgroup. The current review focusses on the meaning of anchor statements and argues that when determining the required standard of performance it is more appropriate to consider: (1) what it is important to achieve, and not how difficult it is to achieve it; (2) what all candidates should achieve, and not what a subgroup of candidates would achieve. In summary, current practice should be refined by using an anchor statement which refers to estimating the ‘minimum acceptable performance by every candidate’ for each item being tested, and then requiring each judge to score the relevant aspects of importance which could then be combined to derive a cut-score.

## Introduction

The method of standard setting originally proposed by Angoff in 1971 asks expert judges to estimate item-level performance for a given group of candidates as the basis for deriving a cut-score. Variations of this method are widely used internationally to set cut-scores for the highest stakes assessments (e.g. Impara & Plake, 1997; Downing et al., 2006; General Medical Council, 2014). Much has been published on factors affecting the process, but comparatively little of this work focusses on the key fundamental element i.e. the anchor statement or starting point - notably identified by Impara and Plake (1998). In the standard setting process, the anchor statement directs the judges to focus on a group of candidates and provide performance estimates for them. For example, judges might be directed to ‘Imagine a borderline student’, and make judgements of their performance. Given the importance of the anchor statement in providing the foundation for the quality of the process, we are concerned that there is excessive variation in both the statement and its interpretation, which can be explicitly subjective. We seek to challenge, logically, unqualified acceptance of whichever statement is presented for use as this cornerstone. Following this, we hope to provide reasoned justification for more careful consideration of the Angoff process foundations. Our objective is to determine the most appropriate anchor statement, and provide guidance on its interpretation and application, with the aim of improving consistency and robustness in the process and thus comparability of standards across all applications of the Angoff and its variants.

## The Angoff method of standard setting

The purpose of standard setting is to determine the cut-score (or pass mark) for a test ( Friedman Ben-David, 2000). The Angoff method depends on a panel of expert judges expending much time estimating the difficulty of each item in turn, with the combined average rating becoming the cut-score for the assessment ( McKinley & Norcini, 2014). The process depends fundamentally on what is actually being considered when attempting to determine the student performance on each item, and the only basis usually provided for this is an anchor statement such as ‘imagine the performance of a minimally acceptable candidate’ as discussed below. The cut-score is intended to represent the required minimum threshold standard, for this hypothetical scenario, that must be met in order to progress or be eligible for an award. The basic premise pivots on the conceptualisation of a specific group of candidates delimited by an explicit anchor statement in order to decide on a cut-score that is empirically justified and not arbitrarily determined.

## Factors affecting the method

The staff resource required for the Angoff method (and its derivatives) exceeds that needed for any other method. Perhaps it is the investment in expert time and the associated implication of scrutiny that has led to the method being widely accepted as the most defensible ‘gold standard’. Extensive simulation modelling has revealed that cut-scores are most precise if panels contain 15 judges comprising a mixture of experts and non-experts, with a second round of discussion and review making little difference to cut-score precision ( Shulruf, 2016). It is possible that judges may be influenced by having to take responsibility for potential consequences, either in being required to justify any outlying judgements, or in managing candidates who don’t meet the required standard. Furthermore, subtle differences in anchor statements persist and provide opportunity for differing interpretations of terms, such as ‘would’ and ‘should’, an effect which has long been firmly established in other fields (e.g. Loftus and Palmer, 1974). Given all of these potential confounders, it is possible that some staff still find it difficult to conceptualise the group specified by the anchor statement and their performance.

A natural corollary of the assertion that staff cannot unanimously agree upon or conceptualise consistently the target group of candidates being evaluated is that their evaluations of item-level performance will vary, at the least in part to them evaluating different groups. Similarly, differential impact of factors such as responsibility for the test implies that each judge has a different goal in the setting of the standard and that this would undermine consistency of approach.

## Anchor statements

In Angoff’s (1971) original suggestion, each judge would be required to “state the probability that the minimally acceptable person would answer each item correctly” and was described in detail by Cizek and Bunch (2007). A review of the published literature for anchor statements used with Angoff methods reveals some variability in practice. While it is usual to require judges to estimate the proportion of ‘candidates’ that would answer each item correctly, those candidates may be minimally ‘acceptable’ ( Plake & Cizek, 2012), ‘competent’ ( Impara & Plake, 1998; Boursicot & Roberts, 2006; Shulruf et al., 2016), ‘proficient’ ( Clauser et al., 2009; Margolis et al., 2016), or ‘qualified’ ( Wheaton & Parry, 2012). For example: the authors own institution until recently used “Imagine a group of 100 minimally competent graduates and ask yourself how many of these candidates would answer the item correctly”, where: “A minimally competent graduate is one who meets the standard by the smallest possible margin. They have enough of the requisite knowledge and are considered to be a safe practitioner although they may be lacking in some areas of expertise.” Although this could become a circular argument; the minimally competent candidate is one who meets the standard by the smallest possible margin; meeting the standard by the smallest possible margin defines the minimally competent candidate. The UK Medical Schools Council national standard setting group recently used ‘What proportion of just safe F1 doctors would get the question correct?’ Identifying the ‘cut’ thus depends on conceptualising the difficulty of the task for candidates who are: just passing, just safe, borderline (implying a range in performance close to the passing threshold and thus differing standards), minimally competent (only just able to perform the task), or proficient (can be entrusted to perform in practice). Competency and proficiency typically apply to skills, so the appropriateness of these terms for knowledge-based assessments may lead to further variability in judgements unless their use is specifically qualified. The use of potentially vague terms such as ‘borderline’ which implies a range of performance and for which there is no clear conceptual consensus of a threshold will further increase variability in the judgements made by different standard setters.

## Anchor statements in relation to aims

The aim of the standard setting process is to derive a cut-score which represents the required minimum threshold that must be met for a particular assessment. If standard setting judges are influenced by student ability ( Taylor et al., 2017) as well as their knowledge of the specific students’ curriculum, and in particular cued to this variable through the anchor statement’s use of terms such as ‘minimally acceptable candidate’, then the implication is that judges are conceptualising how a typical candidate in that position would most likely perform, rather than what is an acceptable performance for a given question and the requirements of the award for which the assessment is part.

If the standard is based on what a minimally acceptable candidate would achieve, then this could be defined by performance data; making judgments about the ability of candidates relative to past candidates who have narrowly achieved the standard. Setting aside the aforementioned issues in conceptualising a particular group of candidates, the judgements depend on the judges’ previous experience of the performance of minimally acceptable candidates and what factors the judge thinks will affect the performance of a typical minimally acceptable candidate, such as the equivalence in delivery of the syllabus relative to the questions being asked. Furthermore, it has been shown that candidate membership of the borderline group based on performance varies considerably, for example across different domains of knowledge and between different clinical skills stations ( Homer et al., 2017). Indeed the borderline group seems to be defined by the tendency to perform satisfactorily on some tasks but not on others. This case specificity has been known about for years of course but rather than try and iron it out perhaps we should go in search of it as the characteristic we need to define a borderline group.

## Standard setting judges’ perception of difficulty for candidates

When considering how a minimally acceptable candidate approaches answering questions, consideration can turn to the perceived level of difficulty of the question itself. Determining question difficulty will have a subjective component. This subjective decision is likely to be based on several factors including the familiarity of the person making the judgement with the subject area ( Clauser, Hambleton & Baldwin, 2016); whether it is felt that this is a piece of knowledge that needs to be known by a candidate at that particular point in time; whether it is peripheral knowledge that only an above average candidate is likely to be cognisant of, or if it is based on a fundamental topic which is covered multiple times in the curriculum. These factors will also vary with the purpose of the test; for example, whether it is formative; designed to establish minimal safe practice; allows admittance or progression to another programme, or identifies excellent performance (e.g. honours).

Examples of this subjectivity might include specialists being experts in their area expecting all candidates to know the answer to a question in their speciality which other specialists or generalists would struggle to answer. Does the question relate to a “red flag” issue that all candidates need to be aware of even if topic exposure in the curriculum is limited (e.g. rare but critically important)? Questions covering topics in areas of more peripheral knowledge might be more difficult to rate in terms of how minimally acceptable candidates might answer. Such candidates might absorb themselves in regurgitating facts that may help them in answering questions involving need-to-know facts and figures to answer the question, but not being able to apply their knowledge. These issues are closely linked to the nature of the test as well. For example, when used in a progress test situation ( van der Vleuten, 1996), designed to assess relative novices on the knowledge they will be expected to have at their final test occasion, how should this discrepancy be factored into item-level performance judgements, or a cut-score based on the final level adjusted for candidates at earlier stages of development ( Ricketts, Freeman, & Coombes, 2009)?

Other factors that might be considered in rating a question’s difficulty are: how complex the question is, the length of the question, the detail of information included in the question, and if it is felt that the information covers threshold concepts that candidates traditionally struggle with. Complex questions might require a candidate to apply their knowledge to work out an answer to the first part of a question and then use this to answer the second part of a question. Minimally acceptable candidates may either not have the knowledge base to answer the question, they may struggle to apply more than one piece of information, or they may not have sufficient depth of understanding to be able to apply knowledge more widely. Questions that are straightforward to answer but contain a large amount of text, data, or information in tables may be perceived as more difficult by such candidates. Similarly, topics that candidates may not feel are important might be perceived as more difficult simply due to a lack of familiarity with the subject area. There may also be some questions that a limited knowledge base makes answering easier, as candidates will not over-analyse the question or response options, or be less likely to misapply or over-generalise knowledge they do possess. Questions may be presented in a manner unfamiliar to a candidate and this might make some candidates less likely to attempt the question (e.g. data being presented in a graph rather than described using words to show a key principle). There are also questions that need transfer of knowledge across topic areas, where candidates might be familiar with a concept but unable to apply it to a new situation. Consideration of these factors may influence the interpretation of difficulty to varying extents by different judges. Thus the variety of influencing factors is only part of the issue. Not only are the anchor statements likely to be interpreted in different ways by different judges, but each judge will also give different weights and consideration to each of these factors when making a judgement about members of the target population.

## What does minimally acceptable mean?

The difficulty in achieving the cut-score varies for candidates of different ability. Only those candidates who achieve close to the cut-score can be said to have an ability that corresponds to the cut-score, but these candidates are defined by the outcomes of the test and therefore cannot be identified in advance and used to determine the cut-score for the test (the aforementioned circular argument). Thinking about what any particular sub-group of candidates might achieve under test conditions is a potential distraction.

How taking a test affects the performance of any particular sub-group of candidates should not influence the standard. Conceptualisation of how difficult a question might be for a minimally acceptable candidate is challenging, particularly for those judges who have not taught the topic to the candidates. While the use of Yes/No judgments has been incorporated as a way to reduce cognitive load ( Impara & Plake, 1997; Cizek, Bunch & Koons, 2004), the inconsistent semantics and pragmatic context in anchor statements continues to plague judgements. Judgement of difficulty becomes further clouded by issues such as differences in attendance, learning style, and use of additional learning support. Whether a candidate finds it easy or hard should have no bearing, they either meet the minimum standard required or not. The focus should be on contemplating the standard required for ***all*** candidates. Thus it is the *importance* of the content for the standard, and not how *difficult* it is (how candidates are expected to perform), that matters. Experts should not be trying to second guess what a ‘just good enough’ candidate *would* know, but should be making academic judgements about what *all* candidates *should* know.

Whilst we urge that the importance of question content, and not its perceived difficulty, should form the basis of judgements in the Angoff process, we acknowledge that in some situations perceptions of difficulty may influence judgements of importance. For example, variations in the format of the question presentation (such as amount of text or complex integration of language and data discussed above) may be perceived by judges as affecting its *difficulty*, and those judges subsequently adjusting their rated *importance* to compensate for these variations. This undesirable variation can be overcome to a large extent by requiring every question to adhere to the same style guidelines, but can also be scrutinised at pre-test by the judges, ensuring particularly long or unusual question formats are not disproportionately associated with a particular area of knowledge.

Candidate performance relative to a standard set value can inform staff teaching, but candidate performance should not influence standard setting judgements. Dissociating judgements of item-importance from candidate-performance goes some way to safeguarding this important distinction. Standard setting judgements should be about what the candidate ***needs to know*** to progress and not norm-referenced to what they (or rather, their cohort) know in actuality. If standard setting judgements are about what candidates should know, then the judges are in effect judging the importance of the knowledge contained in the question and this may involve prejudice about the value of the discipline. This could lead to competitiveness between judges in order to maintain the position of their specialism within a discipline.

## Practical implications

If the standard is based on the minimum acceptable knowledge required to progress, then this is what a candidate with the smallest possible margin above the cut-score is required to achieve (i.e. what they should get, in order to pass). The judges must then focus on making value judgements about the importance of the content of the questions. Clearly, the standard depends not on what proportion of candidates should get the item correct, but what proportion of content all candidates should get correct, to meet the minimum standard required. A judgement of what a candidate should know is not necessarily ‘all or nothing’ for any given item, as it may be reasonable to require a question to be only partially answered; or for example to be able to eliminate a certain number of distractors within a multiple choice question, in effect considering how important it is for a candidate to be able to distinguish between the alternative possible answers. Thus the anchor statement becomes “estimate the minimum acceptable performance by every candidate for each item”. In other words, what is the proportion of each item, based on its importance, that every candidate should be able to get correct.

In order to judge what is acceptable in terms of importance, the judges would need to be experts on the stage being assessed, through a thorough appreciation of how the questions relate to the intended learning outcomes. To judge importance, the degree of authenticity of both the item and test conditions must be taken into account. When thinking about meaning, the relative importance of item content could, for instance, be indicated by use of indicative learning outcomes and giving precedence according to the extent that different aspects of content are a prerequisite for other outcomes, and have impact in practice either by being frequently needed or by having a disproportionate individual consequence. For example, a concept could be considered key to understanding several other points, used daily in the workplace, and pose significant risk if got wrong.

To ease the cognitive load, the method could be modified, for example, to separately rate the different aspects of importance that are relevant to the particular programme and stage. For example, links, frequency, and consequence (as suggested above) could be separately rated on a scale, for example, of 0/1/2. Conceptually the scale would represent marginal/important/essential, similar to the relevance-related component in the Ebel ( Ebel, 1972) and Bookmark methods ( Lewis et al., 1999) but without the problematic ‘difficulty’ component. These could be tailored to suit the particular discipline, and include for instance clinical or practical relevance, pedagogical value, or even the relationship to the hierarchy of knowledge ( Bloom et al, 1956).

Extending this example, there would be three ratings of importance for each item and correct answer each rated on a three-point scale, regardless of the difficulty of distractors. When there are two near identical questions, which only differ in terms of their distractors, one may well be more difficult than another. This can be accounted for by considering how important it is to be able to rule out those different distractors. The individual ratings from different judges could then be combined and the mode reported separately as an agreed group rating for each aspect of importance. The agreed group rating for each aspect could be weighted (equally, or in proportions appropriate to the context) with the other ratings of importance for that item and then summed to give a minimum standard (or index of importance) for that item from 0-100%. The standard for every item could then be averaged to derive a final cut-score for the test as a whole. This approach shifts the focus from candidate-performance to item-importance. The method could, used in different ways, permit unrestricted compensation between items, mandate a minimum performance for every item, mandate a minimum performance for selected (highest stakes ‘red flag’) items, or identify certain rating values to indicate candidates who require remediation. The focus on importance could also facilitate multiple cut-scores for different grades within a test.

## Take Home Messages

When articulating anchor statements with a focus on importance, for use in setting the standard of either knowledge or skills based tests:

• Consider what all candidates **should** achieve in order to meet the required standard, and not what a subgroup of candidates **would** achieve. The cut-score needs to reflect what is important to meet the required standard and not how difficult it is to achieve it.
• Terminology which implies a **range** (such as ‘borderline’) should **notbeused** when determining a **cut-score**. Although a range constructed around a cut-score is acceptable where uncertainty needs to be accommodated, it should not feature in the setting of the cut-score.
• Weighting within the standard setting method should be used to **minimise compensation**, to ensure that a candidate cannot pass a test by applying limited specialised knowledge.
• All judges should have detailed knowledge of at least some aspects of the performance required at the stage being assessed, in order to be able to apply **rigour** to the anchor statement.
• Judges should be required to **justify alldecisions** to minimise the potential for bias.

Taking the above into account, a generic anchor statement meeting these recommendations could be “estimate the minimum acceptable performance by every candidate for each item”, and then requiring each judge to score the relevant aspects of importance which could then be combined to derive a cut-score.

## Notes On Contributors

Dr Steven Ashley Burr is an Associate Professor and Deputy Director of Assessment for Medicine at Plymouth University Peninsula Schools of Medicine and Dentistry.

Dr Daniel Zahra is a Senior Assessment Psychometrician at Plymouth University Peninsula Schools of Medicine and Dentistry.

Professor John Cookson is an Emeritus Professor at Hull York Medical School where he was previously Foundation Professor of Medical Education and Undergraduate Dean.

Dr Vehid Max Salih is an Associate Professor (Reader) and Deputy Director of Assessment for Dentistry at Plymouth University Peninsula Schools of Medicine and Dentistry.

Dr Elizabeth Gabe-Thomas is a Senior Assessment Psychometrician at Plymouth University Peninsula Schools of Medicine and Dentistry.

Dr Iain Martin Robinson is an Associate Professor (Reader) at Plymouth University Peninsula Schools of Medicine and Dentistry.
