# Supplementary material for: Angoff anchor statements: setting a flawed gold standard?
Source: MedEdPublish (2016). 2017 Sep 21;6:167. [Version 1] doi: 10.15694/mep.2017.000167 (PMC10885297; doi:10.15694/mep.2017.000167)
Supplement: Peer review history

# Reviewer report: Reviewer response for version 1

**DOI:** 10.21956/mep.19343.r28266

**Published:** 2017-09-24

**Authors:** Trevor Gibbs

**Affiliations:** 1 AMEE

This review has been migrated. The reviewer awarded 3 stars out of 5 An interesting paper that covers a very important but difficult issue. As we progress from what was accepted as an acceptable method of defining the cut score in specific methods of assessment, we often find that discussions expand on what we really mean. The Angoff method is a very useful activity provided that time is given to define the borderline student and that the process is taken through in the correct way, by the correct number of valid assessors and given the correct amount of time to be performed correctly.I think, as expressed by my co-reviewers, that the approach taken here needs to be run through either as a pilot or in a mock activity to expose its true value

Reviewer Expertise:

NA

No decision status is available

# Reviewer report: Reviewer response for version 1

**DOI:** 10.21956/mep.19343.r28265

**Published:** 2017-09-23

**Authors:** Alan Jaap

**Affiliations:** 1 University of Edinburgh Medical School

This review has been migrated. The reviewer awarded 4 stars out of 5 The authors provide both a useful review and thought-provoking perspective on standard setting for high stakes assessments using the Angoff approach. The main focus of this paper relates to the ongoing thorny issue of conceptualising the just acceptable candidate performance. A novel standard setting strategy based on importance of item content, with similarities to Ebel or Bookmark, is proposed as an alternative. This new strategy seems quite complex and it will be interesting to see how it works in practice in due course. It may be that standard setters will experience similar problems in defining and conceptualising importance.In reviewing this article I am reminded of the bottom line that there is no ideal standard setting methodology: each has its own strengths and weaknesses. While the authors express concerns about variability in Angoff judges scores for specific items, others would argue that this is actually a particular strength of this methodology, potentially capturing a rich perspective on many of the issues discussed in this paper, such as item difficulty, importance and question complexity. The key issue for Angoff or Ebel is in the selection of an appropriate group of judges, with both the literature and experience suggesting that diversity in general and expertise in particular are important for consistency of decision making (e.g. Sulruf et al, as referenced by the authors). The authors may wish to consider how they can encompass this diversity with the narrower subject expertise needs suggested by the requirement to determine item importance.Finally, the wording of Angoff anchor statements has evolved iteratively over the years meaning that we are now much closer to achieving consensus on these for particular contexts (e.g. Medical Schools Council Assessment Alliance and graduating medical students in the UK). Ultimately this may prove to be more fruitful than switching to a different type of anchor statement.

Reviewer Expertise:

NA

No decision status is available

# Reviewer report: Reviewer response for version 1

**DOI:** 10.21956/mep.19343.r28264

**Published:** 2017-09-22

**Authors:** James Fraser

**Affiliations:** 1 Griffith University

This review has been migrated. The reviewer awarded 4 stars out of 5 Standard setting is a vexing process for many of us and this paper makes a contribution to ongoing discussions on how to set a cut score that identifies the correct students to progress to the next stage of training, while being equitable and defensible. The authors describe the challenges experts have with the concept of the just good enough student. By proposing a modification of the Ebel/Bookmark methods the paper suggests that the judgement moves from a minimum level of knowledge or competency to a judgement on the importance of a piece of knowledge in an assessment task. In an aside if topics aren’t important should we expect students to know them at all and should we assess them?It would be interesting to hear of a pilot of this project as I wonder if the concept of importance between the expert panel will vary in a similar manner to the concept of just good enough. I also wonder how question construct will affect this as a student may perform differently on questions on the same topic, that presumably have the same importance, but with different question constructs. I thank the authors for an interesting thought provoking paper.

Reviewer Expertise:

NA

No decision status is available
